# Architecture- and Composition-Controlled Self-Assembly of Block Copolymers and Binary Mixtures With Crosslinkable Components: Chain Exchange Between Block Copolymer Nanoparticles

**DOI:** 10.3389/fchem.2022.833307

**Published:** 2022-02-23

**Authors:** Panpan Li, Jesse L. Davis, Jimmy W. Mays, Xu Wang, S. Michael Kilbey

**Affiliations:** ^1^ Shenzhen Research Institute of Shandong University, Shenzhen, China; ^2^ National Engineering Research Center for Colloidal Materials, School of Chemistry and Chemical Engineering, Shandong University, Jinan, China; ^3^ Department of Chemistry, University of Tennessee, Knoxville, TN, United States; ^4^ Department of Chemical and Biomolecular Engineering, University of Tennessee, Knoxville, TN, United States

**Keywords:** block copolymers, self-assembly, polymer chain exchange, soft nanoparticles, crosslinking

## Abstract

Chain exchange behaviors in self-assembled block copolymer (BCP) nanoparticles (NPs) at room temperature are investigated through observations of structural differences between parent and binary systems of BCP NPs with and without crosslinked domains. Pairs of linear diblock or triblock, and branched star-like polystyrene-poly(2-vinylpyridine) (PS-PVP) copolymers that self-assemble in a PVP-selective mixed solvent into BCP NPs with definite differences in size and self-assembled morphology are combined by diverse mixing protocols and at different crosslinking densities to reveal the impact of chain exchange between BCP NPs. Clear structural evolution is observed by dynamic light scattering and AFM and TEM imaging, especially in a blend of triblock + star copolymer BCP NPs. The changes are ascribed to the chain motion inherent in the dynamic equilibrium, which drives the system to a new structure, even at room temperature. Chemical crosslinking of PVP corona blocks suppresses chain exchange between the BCP NPs and freezes the nanostructures at a copolymer crosslinking density (CLD) of ∼9%. This investigation of chain exchange behaviors in BCP NPs having architectural and compositional complexity and the ability to moderate chain motion through tailoring the CLD is expected to be valuable for understanding the dynamic nature of BCP self-assemblies and diversifying the self-assembled structures adopted by these systems. These efforts may guide the rational construction of novel polymer NPs for potential use, for example, as drug delivery platforms and nanoreactors.

## Introduction

Self-assembly of amphiphilic block copolymers (BCPs) has been viewed as a scalable and robust method for the fabrication and engineering of nanomaterials (Ozin et al., 2009; Bates et al., 2012; Gröschel et al., 2013; Jia et al., 2020; Liu et al., 2020; Kang et al., 2021). The spontaneous generation of structurally well-defined soft nanoparticles (NPs) from polymeric building blocks is fundamental to a variety of technologies, including therapeutic encapsulation and delivery (Wang et al., 2017; Zhong et al., 2018), templated synthesis of nanomaterials (Yan et al., 2019; Su et al., 2020), and nanofabrication of patterned surfaces for microelectronics or for catalysis (Ozin et al., 2009; Yan et al., 2015; Ghoshal et al., 2016; Yan et al., 2017). Although the structure of self-assembled NPs created from amphiphilic BCPs is governed by a balance between stretching energy of well-solvated corona chains, packing of the solvophobic chains in the core, and interface formation (Zhulina and Borisov, 2012), it is known that self-assembled NPs are dynamic in nature (Owen et al., 2012). A dynamic equilibrium always exists in an equilibrated system of self-assembled BCP NPs, wherein the NPs can exchange polymer chains with one another (Haliloǧlu et al., 1996; Denkova et al., 2010). Previous studies on chain exchange behaviors of self-assembled BCP NPs have mainly focused on the simple linear diblock copolymer systems (Procházka et al., 1991; Haliloǧlu et al., 1996; Smith and Liu, 1996; Choi et al., 2010; Choi et al., 2011; Lu et al., 2013; Wang et al., 2020).

In terms of diblock copolymers, Mattice and coworkers described three distinct types of exchange mechanisms, which are 1) chain insertion/expulsion, 2) micellar merger/splitting, and 3) micellar spanning (Haliloǧlu et al., 1996). However, simple linear copolymer architectures usually lead to spherical self-assembled structures, which can make it difficult to directly observe chain exchange between similar BCP NPs. Common methods used to investigate chain exchange kinetics of self-assembled BCP NPs include spectroscopic techniques, such as fluorescence quenching (Procházka et al., 1991; Smith and Liu, 1996), or small-angle neutron scattering (Choi et al., 2010; Choi et al., 2011; Wang et al., 2020). In addition, microscopy is also used, and it offers the advantage of being a direct method capable of visualizing BCP NPs, as well as observing stimuli-responsive structural evolution of BCP NPs (Gröschel et al., 2013; Yang and Du, 2020). Using transmission electron microscopy (TEM) or cryogenic-TEM, the chain exchange-induced evolution of particle sizes in mixed micelles (Zhang et al., 1997; Esselink et al., 1998b), time- or concentration-dependent micelle growth (Esselink et al., 1998a; Kelley et al., 2014), and the dynamics of metal nanoparticle-encapsulated micelles (Li et al., 2019) were observed in model systems of spherical micelles formed from polystyrene-*block*-poly(acrylic acid) (PS-*b*-PAA), PS-*block*-poly(2-vinylpyridine) (PS-*b*-PVP) or polybutadiene-*block*-poly(ethylene oxide) (PB-*b*-PEO) diblock or PEO-*block*-poly(propylene oxide)-*block*-PEO (PEO-*b*-PPO-*b*-PEO) triblock copolymers.

Despite these achievements, direct and accurate observation of chain exchange behaviors in BCP NPs is still challenging because of the lack of morphological diversity in self-assembled BCP NP structures—most often, self-assembly of linear BCPs generates spherical structures. In addition, the inherent dispersity of the micelle size distribution and aggregation of micelles can complicate attempts to observe the structural evolution in these systems. Therefore, a useful strategy may be to use pairs of homologous BCP NPs having distinct structures, such as spherical-cylindrical or solid-hollow NPs. In principle, events such as chain exchange will result in structural changes that can be assessed in a direct fashion.

One way to accomplish this is to make use of architectural and compositional complexity of BCPs that is achieved by the precise control afforded through advanced polymerization methods (Kilbey et al., 2001; Alonzo et al., 2006; Ji et al., 2007). A variety of works have shown that these systems can self-assemble into novel nanostructures by microphase segregation (Rupar et al., 2012; Davis et al., 2016). Using a diverse set of PS-PVP BCPs with architectural and compositional variations, we previously demonstrated that it is possible to access a variety of the self-assembled structures by making binary mixtures (Wang et al., 2014). In addition, it is known that macromolecular architecture and composition seriously affect the critical micelle concentration (CMC) of surfactant-like BCPs, which determines the thermodynamic factors of standard Gibbs free energy, and enthalpy and entropy of micellization in the solvent system (Balsara et al., 1991; Voulgaris et al., 1998; Wang et al., 2014; Davis et al., 2016). Therefore, in a selective solvent and at a given temperature, the thermodynamically-driven motion of polymer chains of BCP NPs is greatly impacted by architecture and composition of BCPs. Thus, we hypothesize that it is possible to utilize binary mixture of BCPs having fine-tuned macromolecular architectures and compositions where the chain exchange between self-assembled BCP NPs is highly active at room temperature. These systems will evolve at room temperature, facilitating the direct observation of structural evolution of BCP NPs by active chain exchange, avoiding the need to heat the BCP systems. Another factor that affects the dynamics of self-assembled BCP NPs is crosslinking of specific domains of BCP NPs, which stabilizes the nanostructures (Saito and Ishizu, 1997; Hayward et al., 2005a; Hayward et al., 2005b; O'Reilly et al., 2006; Wang et al., 2018) and suppresses chain exchange between BCP NPs. Consequently, crosslinked BCP NPs can be used as control systems to verify the occurrence of chain exchange in the corresponding non-crosslinked BCP NPs.

Herein, we describe the self-assembly of architecturally and compositionally diversified PS–PVP BCPs in a PVP-selective solvent mixture (of methanol and THF at 3:1 v/v). Systems consisting of binary mixtures of linear and branched star-like BCPs with decidedly different macromolecular structures and compositions were chosen so that changes in structure could be witnessed by imaging and light scattering methods. The binary systems were blended by different mixing protocols, which facilitates inferences related to how nanostructures evolve in these complex systems. In some cases, the solvated corona domains of the parent BCP NPs were selectively crosslinked before mixing by utilizing the interaction between 1,4-diiodobutane (DIB) and pyridine groups in the PVP segments. Based on the structural differences between the specific linear and star BCP NPs as well as the contrast in the kinetics of chain exchange between crosslinked and non-crosslinked BCP NPs, it is possible to observe the structural evolution of binary BCP NPs induced by chain exchange between two types of BCP NPs at room temperature.

## Experimental Section

### Block Copolymer Solution Preparation and Crosslinking

The diblock, triblock, and branched star-like copolymers having PS and PVP blocks were synthesized via anionic polymerization using all-glass reactors with break-seals. The synthesis of these polymers and their characterization data have been reported previously (Kilbey et al., 2001; Alonzo et al., 2006; Ji et al., 2007) and, therefore, is not repeated here. The polymers were characterized by a combination of size exclusion chromatography, ^1^H NMR spectroscopy, elemental analysis, and multiangle laser light scattering (Kilbey et al., 2001; Alonzo et al., 2006; Ji et al., 2007). We refer to the various BCP architectures using D for diblock, T for triblock, and S for star. PS–PVP BCPs were ultrasonically dissolved in THF at a concentration of 1.0 mg/ml, and then methanol was dropwise added into the BCP THF solution under sonication to prepare a BCP methanol/THF (v:v = 3:1) solution with a final concentration of .25 mg/ml. The BCPs self-assemble into NPs due to the microphase separation. For crosslinking of the PVP corona blocks, DIB at either 10 or 35 mol% relative to 2-vinylpyridine (VP) units was added into the BCP methanol/THF (v:v = 3:1) solution. The chemical crosslinking of PVP with DIB was allowed to proceed for 48 h at 25°C under gentle stirring. After all preparations, the solutions were stored at room temperature for a minimum of 5 days before analysis.

### Mixing Protocols

**SCHEME 1:**
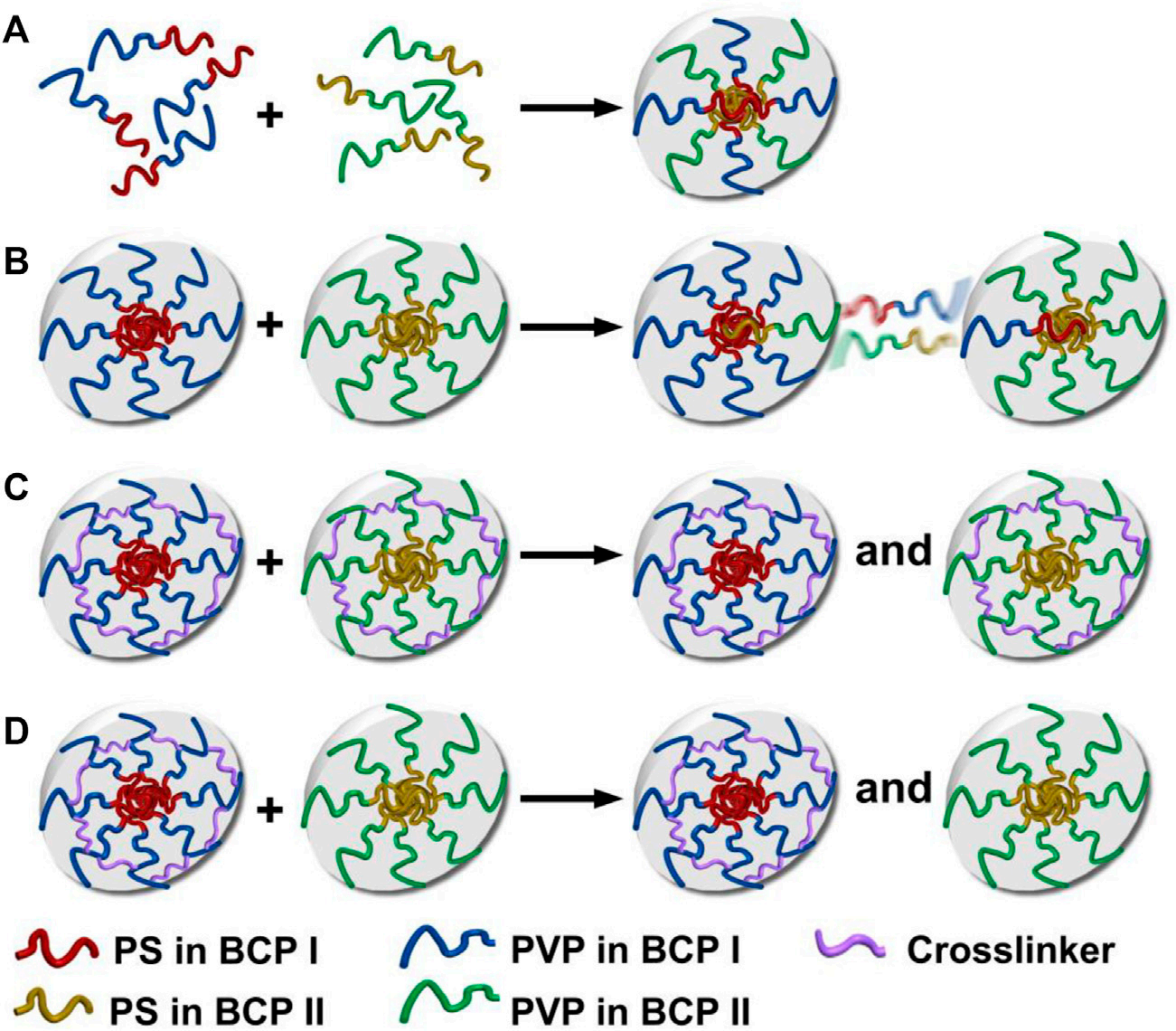
Hypothetical self-assembly and chain exchange for blends created by different mixing protocols: **(A)** premixing of BCPs, BCPs I and II form hybrid NPs; **(B)** postmixing of BCP NPs, where chain exchange occurs between NPs; **(C)** postmixing of crosslinked BCP NPs, where chain exchange between crosslinked NPs is suppressed; **(D)** postmixing of BCP NPs with crosslinked BCP NPs, where chain exchange between crosslinked and non-crosslinked NPs is suppressed.

Four different mixing protocols were used to prepare binary mixtures of two different PS–PVP BCPs at room temperature. These protocols are schematically depicted in Scheme 1. 1) Premixing, where BCPs I and II were separately dissolved in THF at a concentration of 1.0 mg/ml, allowed the copolymers to mix molecularly in a non-selective good solvent, followed by the addition of methanol into the mixture solution to reach a final concentration of *C* = .25 mg/ml. 2) Postmixing involved independently prepared methanol/THF (v:v = 3:1) solutions of BCPs I and II at *C* = .25 mg/ml, which were subsequently mixed together under sonication. Each of these protocols was also applied to cross-linked systems. 3) Postmixing of crosslinked NPs involved combining premade methanol/THF (v:v = 3:1) solutions of DIB-crosslinked NPs made from BCPs I and II under sonication (*C* = .25 mg/ml). 4) Postmixing of crosslinked NPs with non-crosslinked NPs involved mixing methanol/THF (v:v = 3:1) solutions of crosslinked NPs made from BCP I with non-crosslinked NPs of BCP II. Both of these BCP NP solutions were mixed together under sonication at *C* = .25 mg/ml. Following each of these preparations, the solutions were stored at room temperature for a minimum of 5 days before use.

### Self-Assembly on Surfaces

Silicon substrates obtained from Silicon Quest were ultrasonically cleaned successively in toluene, 2-propanol, methanol, and deionized water, each for 15 min, and then immersed in a piranha acid solution (1:3 v/v mixture of 30% H_2_O_2_ and 98% H_2_SO_4_) and heated until no bubbles were released. (Warning: piranha acid is a strong acid and strong oxidizer.) After cleaning, the silicon substrates were dried with a stream of dry N_2_. The presence of silanol groups on the silicon substrate after piranha acid treatment promotes adsorption of BCPs on these surfaces based on the hydrogen bonding between surface hydroxyl and pyridine groups. Silicon substrates were immersed in BCP solutions overnight to allow adsorption of BCPs on surface. The BCP-modified substrates were then rinsed with methanol, sonicated in methanol for 5 min twice, and finally dried under ambient conditions.

### Characterizations

Dynamic light scattering (DLS) measurements were performed using a goniometer-based, four-detector ALV instrument according to our previous reports (Wang et al., 2014; Davis et al., 2016). The hydrodynamic radius (*R*_h_) values reported herein are *apparent* hydrodynamic radii because they are determined at a finite concentration (at .25 mg/ml). For convenience we refer to these simply as the hydrodynamic radius. As an example, a single set of DLS results for sample S1 is presented in Supplementary Figure S1. The light-intensity autocorrelation functions, amplitude distributions, and apparent diffusion coefficient (z-average of the molar mass distribution, which is determined from the normalized mean decay rate, Γ/*q* ^2^ as a function of the angularly dependent amplitude of the scattering wave vector, *q*) (Davis et al., 2016) for other samples are similar to those presented in Supplementary Figure S1. Atomic force microscopy (AFM) images were collected using a Veeco Instruments Nanoscope IIIa multimode atomic force microscope in tapping mode using silicon cantilevers from Applied NanoStructures, Inc. (Mountain View, CA). At least three different areas were imaged on each sample to assess consistency and ensure that the imaged structures are representative. Root mean square (RMS) roughness and particle size analysis of the images were performed using the NanoScope Analysis v140r1sr4 software. Transmission electron microscopy (TEM) images were acquired using a Zeiss Libra 200 MC transmission electron microscope that is equipped with a Gatan UltraScan US1000XP CCD camera. Samples were prepared by drop-casting the polymer solution on a carbon film grid and then staining the dried films by exposure to iodine vapor for 24 h. Images were acquired at the column temperature, which was close to room temperature. The degree of quaternization of the crosslinked BCP NPs was determined via X-ray photoelectron spectroscopy (XPS) using a Kratos Axis ULTRA XPS system with a monochromated aluminum X-ray source. The binding energy scale was corrected to aliphatic carbon at 285.0 eV. For XPS measurements, the polymer solution was drop-cast on a silicon substrate.

## Results and Discussion

### Self-Assembly of Parent Block Copolymers

In this study, PS–PVP BCPs refer to a series of diblock, triblock, and branched star-like copolymers synthesized by sequential anionic polymerization of styrene and 2-vinylpyridine. The self-assembly behaviors of PS–PVP BCPs were systematically studied as a function of macromolecular architecture and composition. In terms of macromolecular topology, the triblocks have a center PS segment connected to PVP end blocks (PVP-*b*-PS-*b*-PVP), and each of the arms of the stars have a diblock structure, with PS as the first block emanating from the central core and PVP as the second (outer) block. Stars having 8-arms or, on average, 26- and 40-arms were used. The synthesis and characterization of these copolymers were described previously (Kilbey et al., 2001; Alonzo et al., 2006; Ji et al., 2007), and the molecular properties of the BCPs are summarized in Table 1 and Supplementary Table S1.

**TABLE 1:** Properties of PS–PVP BCPs and characteristics of their BCP NPs.

Sample ID	BCP	*M*_w_ (kg/mol)^a^	PDI	S/V	morphology^b^	Diameter of spheres by AFM (nm)^c^	*R*_h1_ (nm)^d^	*R*_h2_ (nm)^d^
D1	PS-PVP	[100-60]	1.08	1.7	S	75 ± 14	46 ± 3	
D2	PS-PVP	[54-14]	1.11	3.9	S, R	76 ± 15	29 ± 2	74 ± 4
D3	PS-PVP	[255-24]	1.06	10.6	S	143 ± 47	88 ± 6	
T1	PVP-PS-PVP	[31.25-62.5-31.25]	1.40	1	S	46 ± 18	31 ± 3	
T2	PVP-PS-PVP	[12-96-12]	1.20	4	S, R	43 ± 19	21 ± 3	64 ± 5
T3	PVP-PS-PVP	[6.2-124-6.2]	1.20	10	S	85 ± 31	49 ± 2	180 ± 9
S1	[PS-PVP]_8_	[27-27]_8_	1.18	1	S	38 ± 8	19 ± 1	
S2	[PS-PVP]_8_	[42-14]_8_	1.09	3	S	50 ± 13	22 ± 2	
S3	[PS-PVP]_8_	[42-6]_8_	1.23	7	S	88 ± 29	44 ± 4	132 ± 9
S4	[PS-PVP]_26_	[50-50]_26_	1.23	1	S	47 ± 10	25 ± 3	
S5	[PS-PVP]_26_	[102.5-20.5]_26_	1.45	5	S	186 ± 32	140 ± 7	
S7	[PS-PVP]_40_	[53.75-53.75]_40_	1.26	1	S	48 ± 11	38 ± 3	

a Total molecular weight for linear copolymer is the sum of the numerical values in square brackets, and total molecular weight for star copolymer is the product of the summation of the numerical values in square brackets and the number of arms (denoted by the subscript).

b Aggregation morphologies on surface (using S for sphere, and R for rod).

c The uncertainty is one standard deviation.

d Obtained from DLS in a methanol/THF (v:v = 3:1) mixture at *C* = .25 mg/ml. The DLS data is presented as mean ± standard deviation (*n* = 3).

PS–PVP BCP NP solutions (at a concentration, *C* = .25 mg/ml) were prepared using a cosolvent method. The BCPs were first dissolved in THF, a good solvent for both the PS and PVP blocks, and then a selective solvent for PVP blocks, methanol, was added dropwise into the systems to promote the formation of BCP NPs. In the mixed solvent (methanol/THF (v:v = 3:1)), which is selective for PVP blocks, the PS–PVP BCPs generally self-assembled into NPs of different structures based on the different copolymer architectures and compositions. Supplementary Figure S2 shows the hydrodynamic radii, *R*_h_, distributions for the BCPs in a methanol/THF (v:v = 3:1) mixture at a concentration of .25 mg/ml determined by DLS. The *R*_h_ distributions for most BCPs display a single peak, which means there is only one population of scatterer in solution. Two peaks can be observed in the *R*_h_ distributions for samples D2, T2, T3, and S3, which indicates that more than one type of BCP NPs exist in these solutions. The characteristic *R*_h_ values presented in Table 1 are calculated using the Stokes–Einstein relation. As shown in Table 1, the *R*_h_ for BCPs with the same macromolecular architecture generally increase with increasing styrene to 2-vinylpyridine (S/V) ratio, due to the decrease in the content of soluble PVP segments in the BCPs. Additionally, some of the 26- and 40-arm star BCPs (samples S6, S8, and S9) self-assemble into complex macroscopic aggregates and precipitate in the methanol/THF (v:v = 3:1) mixture owing to their multiple arms, large molecular weights and large S/V ratios (Supplementary Table S1).

**FIGURE 1:**
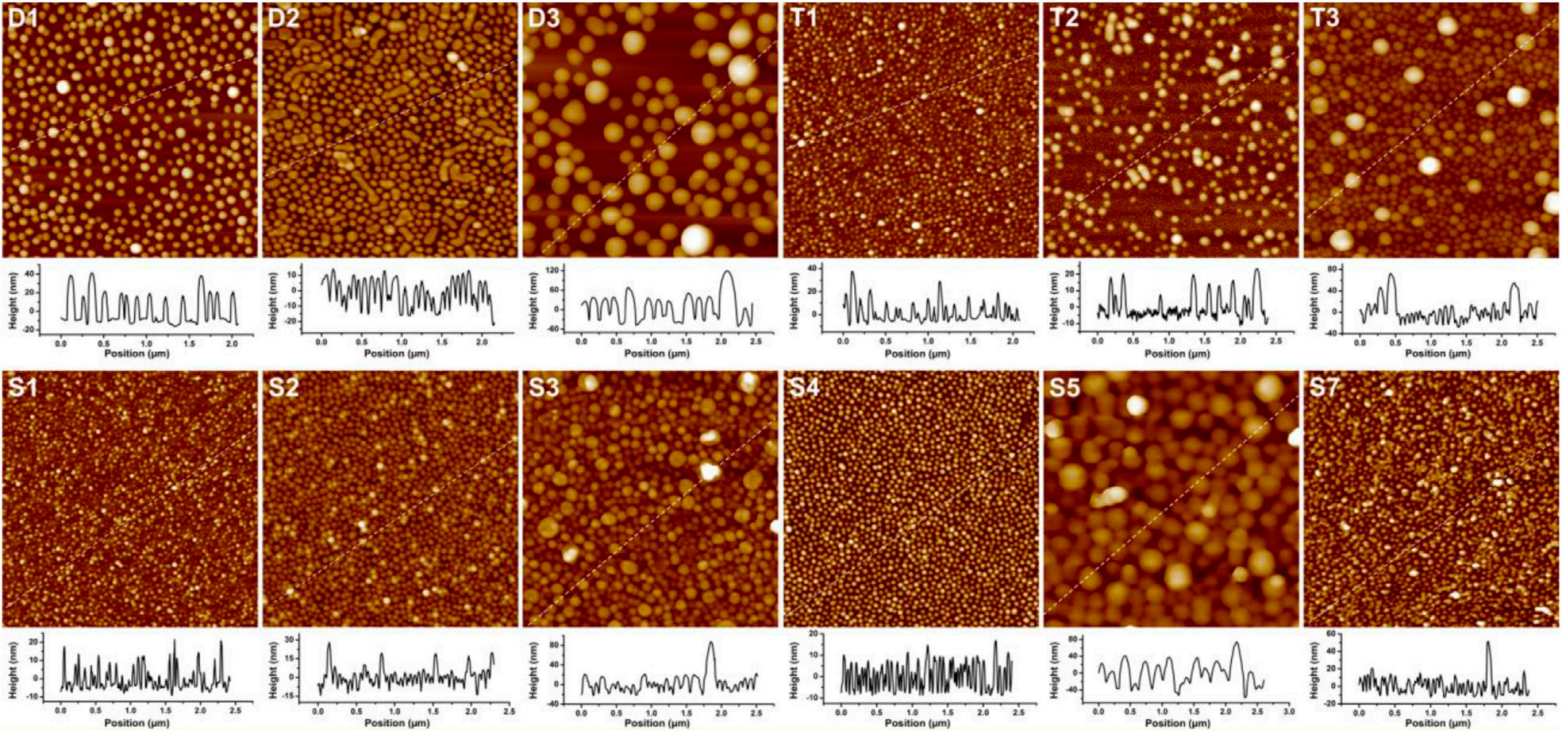
AFM height images (2 μm × 2 µm) and section analyses for linear and star BCPs adsorbed on surfaces. The Z scale of the AFM images is 100 nm for (D1), 60 nm for (D2, T1, T2, S1, S2 and S7), 280 nm for (D3 and S5), 200 nm for (T3), 120 nm for (S3), and 40 nm for (S4). The IDs inset in each panel correspond to the sample IDs used in Table 1.

The adhesion of PS–PVP BCP NPs on surfaces was driven mainly by hydrogen bonding between pyridine groups in the PVP segments and hydroxyl groups on the hydrophilic silicon substrates (Supplementary Figure S3). The resulting films were imaged by AFM, and the representative examples are presented in Figure 1. The AFM topographical (height) images show that the self-assembled NPs for most BCPs are nearly monodisperse spheres, but BCP NPs of other shape are formed in a few samples. Spherical and cylindrical NPs coexist in the films for sample D2, which is in reasonable agreement with the analysis of DLS results indicating that more than one population of scatterers (species with different characteristic sizes) are present. Imaging of substrates exposed to sample T2 revealed two types of spheres with diameters of ∼33 and ∼76 nm, and it appears that a small amount of the larger spheres have merged to form short rods on surface. The BCP NPs present on surfaces for samples T3 and S3 are spherical in shape with highly disperse particle sizes. In all cases, the diameters of spherical BCP NPs on surface were measured by AFM section analysis, as shown in the graphs below the AFM height images in Figure 1. As shown in Table 1, changes in the sizes of the NPs on surface follow trends observed from DLS measurements.

**FIGURE 2:**
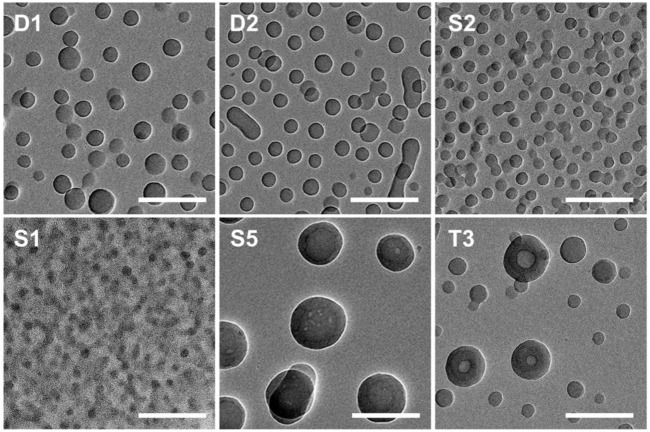
TEM images of BCP NPs cast from a polymer methanol/THF (v:v = 3:1) solution at *C* = .25 mg/ml. Each scale bar is 200 nm. The IDs inset in each image correspond to the sample IDs given in Table 1.

Although it is expected that the structure of BCP NPs seen on the surfaces follows from solution structures, imaging by TEM was also used to gain additional insight into solution structures. Images for representative samples are presented in Figure 2. The self-assembled NPs for samples D1, S1, and S2 appear as uniform objects, suggesting that they are spherical in shape. Sample D2 shows clear evidence of both spherical and cylindrical (rod-like) aggregates. The average diameters for samples D1, S1, and S2 obtained from the TEM images are approximately 55, 30, and 41 nm, respectively. The coexistence of spheres and rods in the TEM image for sample D2 is a good agreement with the AFM and DLS results. The centers of the large BCP NPs (over 100 nm in diameter) formed from T3 are lighter than the periphery, which indicates that these NPs are hollow. The large size of these hollow NPs and electron contrast within the structure suggest that these hollow NPs are vesicles. Vesicular aggregates have attracted much attention because of their potential utility as nanoreactors, nanocontainers, delivery vehicles, and nanofluidic materials (Esselink et al., 1998b; Zhao et al., 2011).

The characteristic sizes and shapes of all of the BCPs studied in the methanol/THF mixed solvent presented in Table 1 demonstrate that the size and shapes of the BCP NPs can be influenced by choosing chain architecture and composition, which provides access to BCP NPs of different structure. In the following sections, we describe how different combinations of BCPs and mixing protocols take advantage of the structural diversity of PS–PVP BCP NPs and greatly diversify the resulting structure of self-assembled aggregates.

### Crosslinking of Parent Block Copolymer Nanoparticles

**FIGURE 3:**
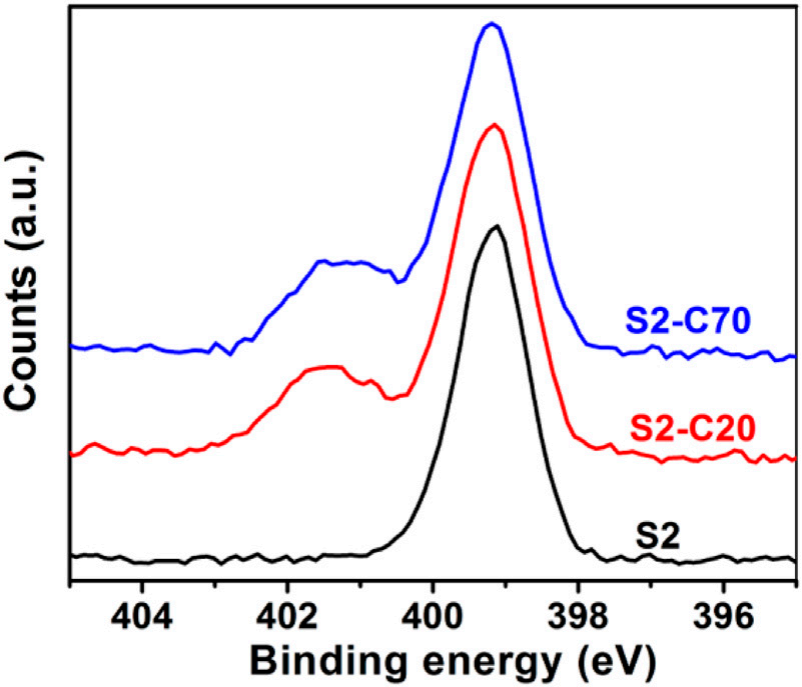
Nitrogen 1s regions of XPS spectra for sample S2, (bottom) non-crosslinked BCP NPs, (middle and top) crosslinked BCP NPs at target PVP CLDs of 20% (middle) and 70% (top).

Crosslinking of PS–PVP BCP self-assembled nanostructures can be achieved by quaternization between pyridine groups in the PVP blocks and DIB within the assembly to afford robust NPs (Saito and Ishizu, 1997; Hayward et al., 2005a; Hayward et al., 2005b). Saito and Ishizu first described the synthesis of flower type microgels by crosslinking of the core-shell micelles formed from PVP-*b*-PS-*b*-PVP BCPs with DIB in solution (Saito and Ishizu, 1997). Hayward et al. (2005a), Hayward et al. (2005b) demonstrated that the DIB crosslinked PS-*b*-PVP BCP films were insoluble in many solvents including acidic water, methanol and THF. These facts suggest that the crosslinking within DIB is feasible for PS–PVP BCP systems. Six kinds of BPC NPs (samples D1, D2, T3, S1, S2, and S5) were crosslinked by DIB at target crosslinking densities (CLDs) of 20% and 70% for PVP blocks by altering the molar feed ratio of alkyliodide to pyridine groups. The amount of DIB added to solution to hit the target CLDs (Table 2) for PS–PVP BCP NPs is calculated from the number of pyridines in the BCPs. The actual CLDs for PVP and PS–PVP BCPs are calculated from the degrees of quaternization in the crosslinked systems, which were determined by XPS measurements. The CLD for the VP groups (denoted as PVP CLD) is the fraction of pyridine groups in the copolymer that are crosslinked, while the CLD for the copolymer (noted as BCP CLD) is the product of the PVP CLD and the fraction of PVP in the BCPs. The XPS spectra for sample S2 at different target PVP CLDs are presented in Figure 3. In the case of non-crosslinked sample S2, a single peak is observed at 399.2 eV (Figure 3, bottom), corresponding to the binding energy of pyridine N 1s electrons. Following crosslinking of BCP NPs via reaction with DIB at target PVP CLDs of 20% (Figure 3, middle, noted as S2-C20) and 70% (Figure 3, top, noted as S2-C70), a second peak corresponding to quaternized pyridine is seen at 401.4 eV, indicating the successful crosslinking of PS–PVP BCP NPs by DIB. The peak area ratio of quaternized to nonquaternized pyridine groups for sample S2-C70 is higher than that for sample S2-C20, which demonstrates that the actual PVP CLD can be controlled by adjusting the feed ratio of DIB to pyridine groups in the PVP chains. The actual PVP CLD is determined from the relative concentrations of quaternized and nonquaternized pyridine groups, as determined by fitting Gaussian curves to the peaks observed in the XPS spectra. Values of the actual PVP CLDs for sample S2 at different target PVP CLDs and for samples D1, D2, S1, S5, and T3 at a target PVP CLD of 70% are listed in Table 2. From these results it is observed that the actual PVP CLD for sample S2 at a crosslink density of 20%, which we designate as S2-C20, is close to the original target (27.7% versus 20% target); however, the actual PVP CLDs achieved at the higher target of 70% are much lower for these BCP NPs. As shown in Table 2, the actual PVP CLDs hardly exceed 39%, which may suggest that achieving high crosslinking densities is limited by steric effects. A more detailed study to probe this phenomenon is deferred to a later effort. Nevertheless and as expected, the structure of the BCP NPs determined after crosslinking by DIB are generally consistent with their original structures. As seen in Supplementary Figures S4, S5 a small number of aggregates due to the crosslinking between several NPs can be observed, which also leads to an increase in values of *R*_h_ of crosslinked BCP NPs for samples D1, D2, S1, and S2 (compare *R*_h_ values between Tables 1, 2). On the other hand, crosslinking of samples T3 and S5 produces a decrease in *R*_h_, indicating that the crosslinking reaction and DIB-based crosslinks change the swelling of the PVP corona. It should be noted that van Hest and coworkers have discovered a crosslinker-induced shape transformation (from spherical to tubular) for polymeric vesicles (“polymersomes”) constructed from BCPs when an excess of crosslinkers exist in the systems (van Oers et al., 2013).

**TABLE 2:** Properties of crosslinked PS–PVP BCP NPs.

Sample ID	Target PVP CLD^a^ (%)	Target BCP CLD^a^ (%)	Actual PVP CLD^b^ (%)	Actual BCP CLD^b^ (%)	Diameter of spheres by AFM (nm)^c^	*R*_h1_ (nm)^d^	*R*_h2_ (nm)^d^
D1-C70	70	26.3	20.1	7.6	78 ± 17	64 ± 4	
S1-C70	70	35.0	31.5	15.8	39 ± 9	26 ± 2	
D2-C70	70	14.4	12.7	2.6	77 ± 16	33 ± 3	91 ± 8
S5-C70	70	11.7	38.8	6.5	187 ± 61	98 ± 5	
S2-C20	20	5.0	27.7	6.9	53 ± 8	25 ± 3	
S2-C70	70	17.5	35.5	8.9	62 ± 11	30 ± 4	130 ± 8
T3-C70	70	6.4	19.3	1.8	93 ± 46	101 ± 12	

a Calculated from the feed molar ratio of DIB to PVP segments or PS–PVP BCPs.

b Actual CLDs are determined from fitting XPS data.

c The uncertainly is expressed as one standard deviation.

d Results acquired from DLS in a methanol/THF (v:v = 3:1) mixture at *C* = .25 mg/ml. The DLS data is presented as mean ± standard deviation (*n* = 3).

### Structural Characterizations of Binary Block Copolymer Mixtures

Though the self-assembly behaviors of fifteen BCPs were studied, BCPs that exhibited similar self-assembled structures were not selected for crosslinking and chain exchange studies because the changes in such systems would not be very evident. Based on that, we selected three binary mixtures, including D1+S1 (large solid spheres + small solid spheres), D2+S5 (small solid spheres + large solid spheres), and S2+T3 (small solid spheres + large hollow spheres). With these choices, the two parent NPs have completely different self-assembled structures, which benefits the effort to examine chain exchange behaviors by directly observing structural evolution as affected by mixing of self-assembled or crosslinked BCP nanoparticles.

**FIGURE 4:**
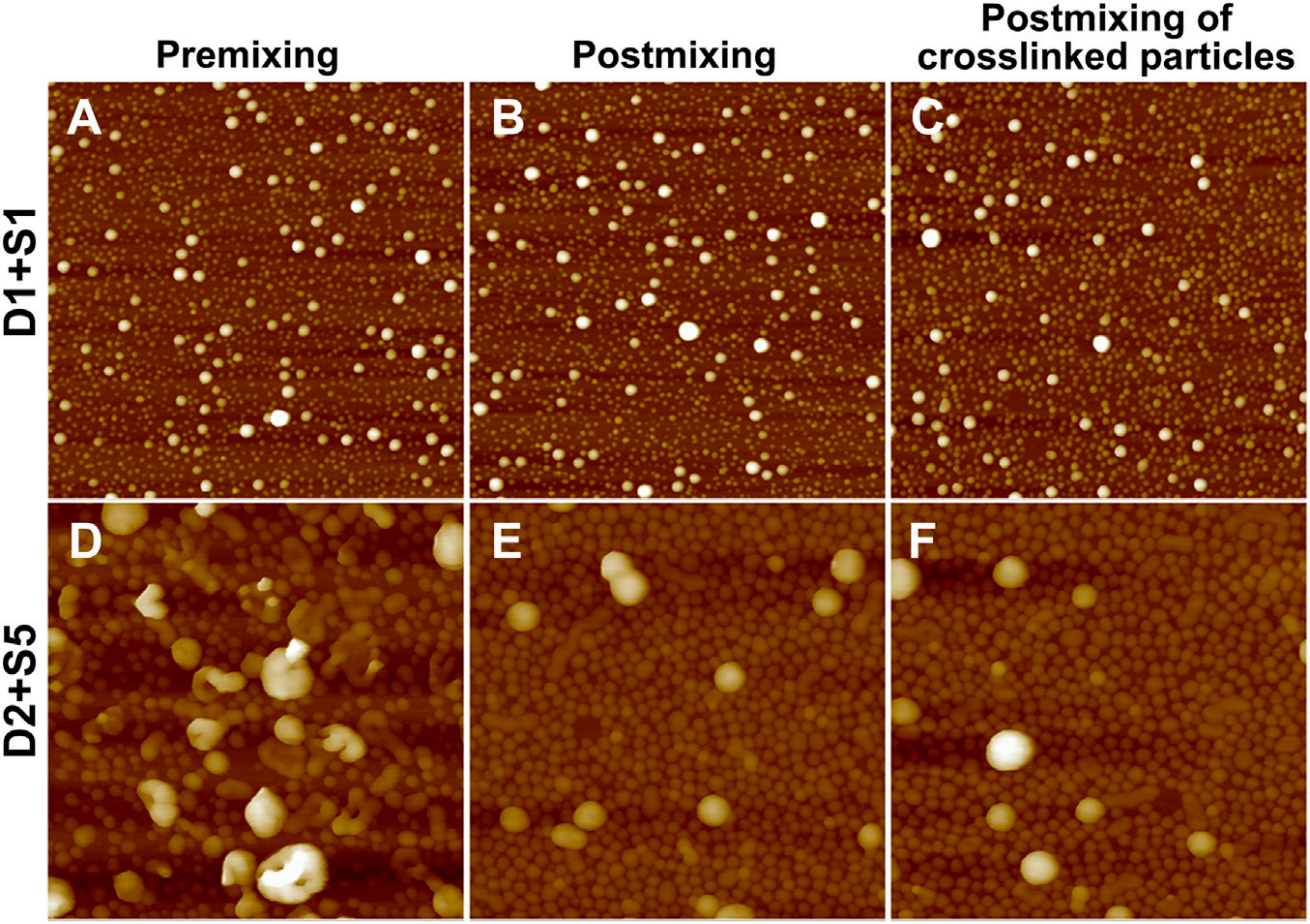
AFM height images (2 μm × 2 µm) of mixtures of **(A)** D1S1-1, **(B)** D1S1-2, **(C)** D1S1-3, **(D)** D2S5-1, **(E)** D2S5-2, and **(F)** D2S5-3. These mixture IDs correspond to those given in Table 3. The Z scale for the images is 80 nm for **(A–C)** and 280 nm for **(D–F)**.

**FIGURE 5:**
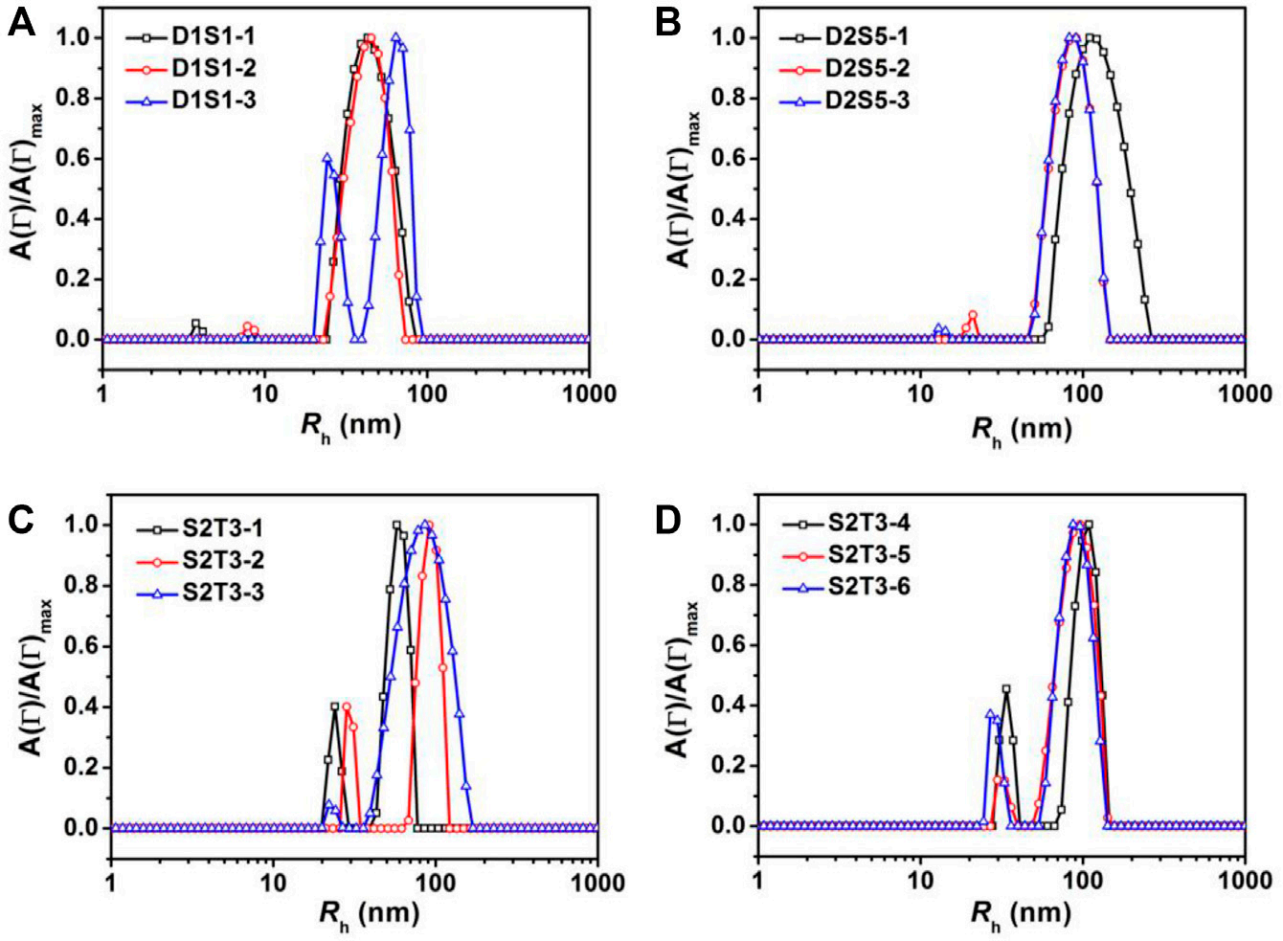
Representative hydrodynamic radii, *R*_h_, distributions for mixtures of **(A)** D1+S1, **(B)** D2+S5, **(C,D)** S2+T3 in a methanol/THF (v:v = 3:1) mixture at *C* = 0.25 mg/ml. The IDs in the figure legends correspond to sample IDs given in Table 3.

Binary blends of linear and star BCPs were prepared either by premixing or postmixing (Li et al., 2006; Yoo et al., 2007) as described in the Experimental Methods section. As described in our previous study, premixing of binary BCP mixtures is a facile strategy to enrich the structures adopted by the BCP NPs because the mixtures principally self-assemble into hybrid NPs, leading to novel nanostructures (Wang et al., 2014). Postmixing of binary BCP NPs is frequently used to study kinetic events such as chain exchange between micelles. In the postmixing strategy, two different types of BCPs are allowed to form NPs, and then the solutions are blended. As a result, the two types of BCP NPs approach equilibrium simultaneously (Choi et al., 2011; Lu et al., 2013). In the studies presented herein, which are schematically depicted in Scheme 1, a protocol of postmixing two different crosslinked BCP NPs was used as a control, as chain motion should be suppressed in this system. Three pairs of BCP mixtures were used to investigate the relationships between the mixing methods and the resulting structures of BCP NPs. These are referred to as D1S1, D2S5, and S2T3. As shown in Figures 4A–C, D1S1 mixtures made by three different mixing methods (denoted as D1S1-1, D1S1-2, and D1S1-3, see Table 3) display nearly identical self-assembled structures on the surface with two types of NPs that are spherical in shape with diameters close to the individual self-assemblies of the parent BCPs. The number of the large NPs (diameter >50 nm) in the self-assembled films observed in Figures 4A–C and their RMS roughness are similar in these three samples (Table 3). The system identified as D1S1-3, which resulted from postmixing crosslinked parent BCP NPs, displays an *R*_h_ distribution with two resolved, narrow peaks located at ∼33 and ∼82 nm (Figure 5A), which correspond to two sizes of NPs observed in the AFM image displayed in Figure 4C. On the other hand, the *R*_h_ distributions for D1S1-1 and D1S1-2 exhibit a single, broad peak centered at ∼47 nm. We attribute this to the similar size of the BCP NPs present in these systems. Although the AFM images acquired for D1S1-2 and D1S1-3 show no obvious differences, the change in *R*_h_ distribution (one peak for D1S1-2 and two peaks for D1S1-3) during the crosslinking process suggests that crosslinking has an impact on the structures resulting from equilibration of the D1+S1 systems. For D2+S5 mixtures, the NP size for the premixed blend (D2S5-1) is larger than that for the postmixed systems (D2S5-2). In addition, the NP shapes for these two blends are different, as reflected in the AFM images acquired from these systems. (See Figures 4D,E and the *R*_h_ distributions in Figure 5B) A comparison of results for the postmixed blend D2S5-2 and the postmixed, crosslinked blend D2S5-3 shows that there is little difference in NP structures (Figures 4E,F), number of large NPs, RMS roughness (Table 3), and *R*_h_ distributions (Figure 5B). Thus, the mixed systems formed from D2+S5 in the methanol/THF solvent mixture appear to not exchange chains at room temperature. As shown in Figures 4B,E, the postmixed NPs identified as D1S1-2 and D2S5-2 seem like simple combinations of the parent BCP NPs, in which the component NPs maintain their original structures. These results indicate that in these systems (D1+S1 and D2+S5 mixtures), chain exchange between aggregates is generally suppressed at room temperature. The exchange of chains in BCP NPs is often suppressed at room temperature due the micelles having “frozen cores,” but chain exchange can occur at elevated temperatures (Yoo et al., 2007; Lu et al., 2013). However, it also has been observed that self-assembly behaviors of BCPs (and BCP mixtures), such as micelle shape and CMC, can change greatly with increases in temperature (Yoo et al., 2007).

**TABLE 3:** Mixing strategy at a 1:1 mass ratio of BCPs and properties of the binary mixtures of PS–PVP BCP NPs.

Mixture ID	BCP I^a^	BCP II^a^	Mixing protocol	Number of large NPs^b^	RMS roughness (nm)^c^	*R*_h1_ (nm)^d^	*R*_h2_ (nm)^d^
D1S1-1	D1	S1	Premixing	>20	7 ± 1	47 ± 6	
D1S1-2	D1	S1	Postmixing	>20	8 ± 1	45 ± 8	
D1S1-3	D1-C70	S1-C70	Postmixing	>20	7 ± 1	32 ± 4	83 ± 7
D2S5-1	D2	S5	Premixing	>20	32 ± 6	151 ± 10	
D2S5-2	D2	S5	Postmixing	10–15	17 ± 3	91 ± 5	
D2S5-3	D2-C70	S5-C70	Postmixing	10–15	19 ± 4	100 ± 8	
S2T3-1	S2	T3	Premixing	5–10	11 ± 2	33 ± 3	74 ± 6
S2T3-2	S2	T3	Postmixing	5–10	8 ± 2	51 ± 7	150 ± 12
S2T3-3	S2-C70	T3-C70	postmixing	>20	20 ± 3	50 ± 4	136 ± 9
S2T3-4	S2	T3-C70	postmixing	5–10	10 ± 1	50 ± 3	152 ± 8
S2T3-5	S2-C70	T3	postmixing	>20	21 ± 4	41 ± 3	138 ± 9
S2T3-6	S2-C20	T3	postmixing	5–10	14 ± 3	42 ± 3	147 ± 11

a BCPs I and II correspond to sample IDs given in Tables 1, 2.

b Number of the large particles in a 2 μm × 2 µm AFM image. For this, large is set at diameters >50 nm for D1+S1 mixtures and diameters >100 nm for D2+S5 and for S2+T3 mixtures.

c Acquired by AFM roughness analysis, and the uncertainty represents one standard deviation.

d Results acquired from DLS measurements in a methanol/THF (v:v = 3:1) mixture at *C* = .25 mg/ml. The DLS data is presented as mean ± standard deviation (*n* = 3).

**FIGURE 6:**
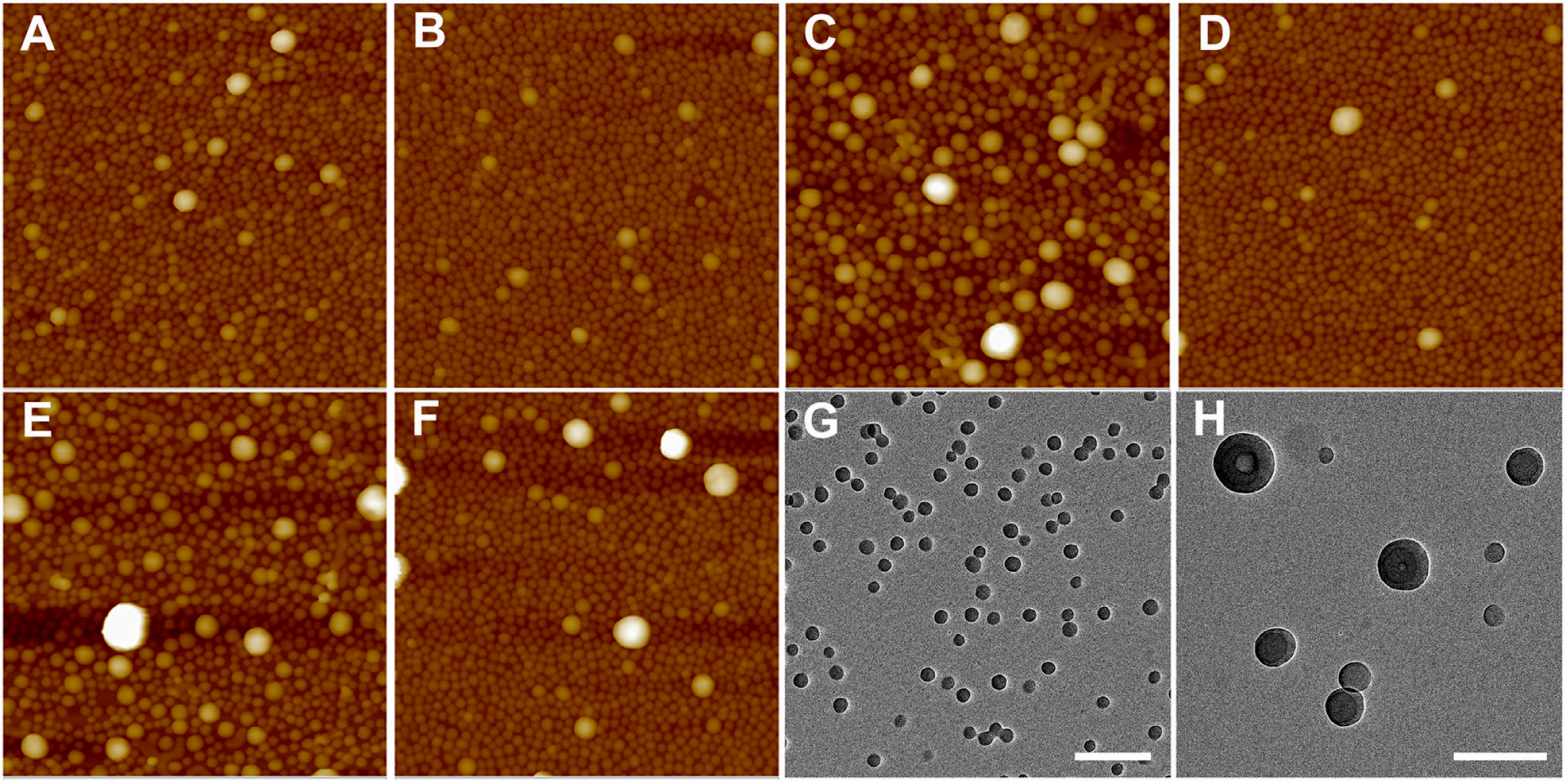
**(A–F)** AFM height images (2 μm × 2 µm) of mixtures of **(A)** S2T3-1, **(B)** S2T3-2, **(C)** S2T3-3, **(D)** S2T3-4, **(E)** S2T3-5, and **(F)** S2T3-6. The Z scale for **(A–F)** is 200 nm. **(G,H)** TEM images of mixtures of **(G)** S2T3-2, and **(H)** S2T3-3. The scale bar in **(G,H)** is 200 nm. The mixture IDs are corresponding to the IDs in Table 3.

Because of the importance of architecture and composition on self-assembly of BCPs, it is possible to choose pairs of PS–PVP BCPs that, when mixed, exhibit clear structural changes upon postmixing, which indicates that chain exchange is active at room temperature. As we noted earlier, in a methanol/THF (v:v = 3:1) mixture, sample S2 self-assembles into solid spheres with an average diameter of ∼50 nm (determined from AFM imaging), while the self-assembled NPs for sample T3 exhibit disperse particle sizes (average diameter ∼85 nm), including hollow NPs having a diameter exceeding 100 nm. When samples S2 and T3 were combined by the premixing protocol (the resulting mixture is noted as S2T3-1), AFM imaging indicates that the system hybridizes into small spheres (Figure 6A). In this case, premixing of S2 and T3 follows Scheme 1A, leading to a molecularly mixed system. The *R*_h_ distribution for S2T3-1 mixture exhibits two adjacent peaks located at approximately 30 and 70 nm (Figure 5C). Both the AFM and DLS results indicate that the particle sizes in the molecularly mixed system are much smaller than that for the parent T3 BCP NP.

As shown in Figure 6B, the BCP NPs on surface made from postmixed S2+T3 mixture (S2T3-2) are spherical in shape. The self-assembled film of S2T3-2 mixture has a small RMS roughness of 8.2 ± 1.7 nm, and only a few large NPs (diameter >100 nm) can be observed in Figure 6B, which indicates that an exchange of polymer chains occurs in this postmixed system, even at room temperature (Scheme 1B). Comparatively, the self-assembled film of postmixed crosslinked S2 and crosslinked T3 at a target PVP CLD of ∼70% (S2T3-3) shows completely different structures: this postmixed system has a large RMS roughness of 20.1 ± 2.9 nm, and a significant number of large NPs (diameter >100 nm) are observed in Figure 6C. Indeed, the large NPs in S2T3-3 mixture originate from the crosslinked NPs of sample T3. This finding illustrates that no obvious chain exchange occurs in the crosslinked system–the two component BCP NPs in the mixture maintain their original structures adopted by the parent BCP NPs (Scheme 1C). Although two components in the mixture have an identical target PVP CLD, the actual BCP CLD for S2 (8.9%) is much larger than that for T3 (actual BCP CLD = 1.8%) due to the smaller S/V value for sample S2 compared to that for sample T3. To elucidate the impact of BCP CLD on the chain exchange behaviors in binary BCP NPs, crosslinked BCP NPs are blended with non-crosslinked BCP NPs by postmxing (Scheme 1D). As shown in Figure 6D, the adsorbed structure resulting from postmixing the non-crosslinked S2 and crosslinked T3 at an actual BCP CLD of 1.8% for S2T3-4 displays similar structure and RMS roughness with that for S2T3-2. This is mainly because the BCP CLD for T3 is too low to “freeze” the structure of the NPs and, therefore, chain exchange between NPs in this binary mixture is not suppressed. In contrast, the chain exchange between binary BCP NPs is suppressed in the postmixed blend of crosslinked S2 at an actual BCP CLD of 8.9% and non-crosslinked T3 (S2T3-5). In this case, the high BCP CLD for S2 (Figure 6E) that prevents chain exchange results in the film exhibiting various structures, several large NPs (diameter >100 nm), and an RMS roughness that are similar to the binary mixture identified as S2T3-3. For comparison, the self-assembled film resulting from the case where S2 is crosslinked at a low actual BCP CLD of 6.9% and combined with non-crosslinked T3 by postmixing (this mixture is noted as S2T3-6) is presented in Figure 6F. The structure of BCP NPs on the film surface for S2T3-6 is similar to that for S2T3-4, but there are more large NPs (diameter >100 nm) in S2T3-6 than in S2T3-4, even though the target BCP CLDs in these systems are similar. We suspect this is because the large size and complex structure of the NPs formed from T3 make chemical crosslinking difficult (Supplementary Scheme S1).

From the transformations observed in the self-assembled structures resulting from mixtures of non-crosslinked T3 and crosslinked S2 at different target PVP CLDs (0%, 20%, and 70% for S2T3-2, S2T3-6, and S2T3-5, respectively), as well as the number of large NPs and the RMS roughness that increase with increasing PVP CLDs, we conclude that the chain exchange behaviors in BCP NPs and the structures of BCP NPs can be manipulated through careful variation in the PVP CLD. As shown in Figures 5C,D, the *R*_h_ distributions for S2T3-2, S2T3-3, S2T3-4, S2T3-5, and S2T3-6 mixtures generally exhibit two constant peaks located at *R*_h1_ ∼30 nm and *R*_h2_ ∼110 nm, which means two types of NPs exist in these systems. (The small size distributions are from either sample S2 or sample T3, but the large ones come from sample T3 exclusively.) Among these binary mixtures, S2T3-3 shows the weakest signal from the scatterers having a size of *R*_h1_ and the broadest peak corresponding to large aggregates (*R*_h2_), suggesting that the main population of scatterers in this crosslinked system are large particles. Furthermore, TEM imaging of the structures of BCP NPs in mixtures S2T3-2 and S2T3-3 indicates that sample S2T3-2 exhibits solid spherical NPs with a uniform particle size of ∼45 nm, but no large hollow NPs similar to those observed in the parent sample T3 are observed (Figure 6G). On the other hand, TEM imaging of S2T3-3 shows large hollow spheres and small solid spheres coexist in the mixture (Figure 6H). This direct evidence further clarifies the occurrence of chain exchange in the postmixed system of non-crosslinked S2 and T3 BCP NPs at room temperature, but chemical crosslinking can effectively suppress the chain exchange between binary BCP NPs. Overall, the AFM and TEM images of S2T3-2 (Figures 6B,G) show no large hollow particles, indicating that the large hollow particles observed from T3 could not maintain their structures when mixed with S2. By comparison, large hollow particles exist in S2T3-3 (Figures 6C,H), which indicates that crosslinking of PVP corona chains inhibits restructuring. We attribute this ability of the non-crosslinked system to reconfigure to be due to chain exchange.

We investigated the polymer chain exchange behaviors by directly observing the structural evolution during the mixing of self-assembled or crosslinked BCP NPs. These studies were enabled by using BCPs with different architectures and compositions, some of which self-assembled into different structures, including spheres, rods, and hollow vesicles. Thus, by imaging we can observe structural changes, which we attribute to chain exchange. Interestingly, the structural change did not occur in (non-crosslinked systems of) D1+S1 and D2+S5, but did occur in the S2+T3 binary mixture. We suspect that the stability of the self-assembled NPs is a key factor that influences the ability to undergo structural changes. As mentioned earlier, the exchange of chains between BCP NPs is often suppressed due the micelles having “frozen cores.” We suspect that this is at play in D1+S1 and D2+S5 systems. Self-assembly of copolymer T3 resulted in the formation of two different types of NPs, namely small solid spheres and large hollow spheres (as seen in Figure 2), which suggests mutability of the large hollow spheres when physically mixed with sample S2. Although these structural changes are attributed to chain exchange between self-assembled BCP NPs, a more detailed study to probe this phenomenon and understand the underlying mechanism is clearly necessary, and these studies are likely to be facilitated due to the structural differences of the architecturally and compositionally complex copolymers.

## Conclusion

We have demonstrated a strategy based on binary mixing of self-assembled PS–PVP BCP NPs formed from architecturally and compositionally diverse copolymers, which leads to different nanostructures. We emphasize that the architectural and compositional complexity of the parent copolymers is important in the effort to diversify the self-assembled structures of BCP NPs formed when linear and star block copolymers are mixed, and also facilitates insights into chain exchange behaviors. We find that specific linear/star BCP pairs postmixed systems exhibit clear structural changes after several days that are allowed for equilibration at room temperature. This investigation which centered on the role of architecture, composition, and mixing protocols on the exchange of chains between differently structured BCP NPs, suggests that it is possible to expand the type (and characteristic size) of nano-objects produced by BCP self-assembly. We believe that this work enables other interesting pursuits, including studies directed at the relation between nanocarrier structures and drug delivery behaviors, and how thermodynamic factors impact the morphologies of BCP NPs created from BCP mixtures, especially copolymer systems relying on self-assembly of architecturally complex copolymers.

## Data Availability

The raw data supporting the conclusion of this article will be made available by the authors, without undue reservation.
