# Supplementary material for: Architecture- and Composition-Controlled Self-Assembly of Block Copolymers and Binary Mixtures With Crosslinkable Components: Chain Exchange Between Block Copolymer Nanoparticles
Source: Front Chem. 2022 Feb 23;10:833307. doi: 10.3389/fchem.2022.833307 (PMC8906501; doi:10.3389/fchem.2022.833307)
Supplement: Supplementary file 1 [file DataSheet1.pdf]

SUPPLEMENTARY MATERIAL

Architecture- and Composition-Controlled Self-Assembly of Block Copolymers and Binary Mixtures with Crosslinkable Components: Chain Exchange between Block Copolymer Nanoparticles

Panpan Li,[1,2] Jesse L. Davis,[3] Jimmy W. Mays,[3] Xu Wang,[1,2]* and S. Michael Kilbey II[3,4]*

[1]Shenzhen Research Institute of Shandong University, Shenzhen, China

[2]National Engineering Research Center for Colloidal Materials, School of Chemistry and Chemical Engineering, Shandong University, Jinan, China

[3]Department of Chemistry, University of Tennessee, Knoxville, TN, United States

[4]Department of Chemical and Biomolecular Engineering, University of Tennessee, Knoxville, TN, United States

*E-mails: wangxu@sdu.edu.cn (X.W.); mkilbey@utk.edu (S.M.K.II)

TABLE S1 | Properties of PS–PVP BCPs and characteristics of their BCP NPs.

sample ID	BCP	M_w (kg/mol)[a]	PDI	S/V
S6	$[PS\text{-}PVP]_{26}$	$[103.8\text{-}11.5]_{26}$	1.36	9
S8	$[PS\text{-}PVP]_{40}$	$[106.25\text{-}21.25]_{40}$	1.16	5
S9	$[PS\text{-}PVP]_{40}$	$[108\text{-}12]_{40}$	1.30	9

[a]Total molecular weight for linear copolymer is the sum of the numerical values in square brackets, and total molecular weight for star copolymer is the product of the summation of the numerical values in square brackets and the number of arms (denoted by the subscript).

Dynamic light scattering (DLS) analysis

Figure S1a shows the normalized light intensity autocorrelation function for sample S1 in a methanol/THF (v:v = 3:1) mixture at a concentration of 0.25 mg/mL. A single decay mode is observed, and the normalized light intensity autocorrelation function begins at a high value (~0.9), which indicates there are a large number of NPs (strong signal and strong correlation at short lag times, τ). The R_h distribution for sample S1 presented as Figure S1b shows a single, sharp distribution centered at R_h ~20 nm, which indicates that there is one population of scatterers in the solution. The plot of Γ/q^2, where Γ is the mean decay rate, shows no q-dependence over the range studied (Figure S1c), which is indicative of spherical particles. A linear fit (red line in Figure S1c) with extrapolation to $q^2 \rightarrow 0$ provides the z-average diffusion coefficient, $\langle D \rangle_z$. This is an apparent diffusion coefficient, as it is measured at finite concentration. For example, for sample S1, we find $\langle D \rangle_z = 2.28 \times 10^{-7}$ cm^2/s, and from the Stokes-Einstein relation, the characteristic apparent hydrodynamic radius, R_h, is $R_h = k_BT/(6\pi\eta$

<D>$_z$) = 19 nm. In all cases we used η = 0.504 cP for the viscosity of the mixed solvent.

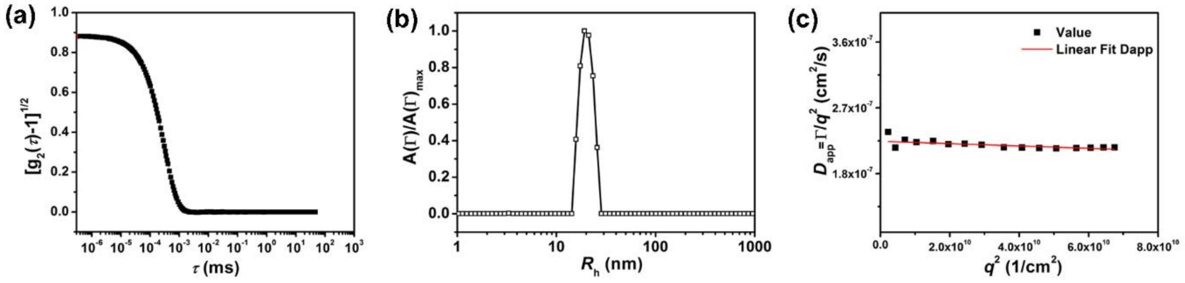

FIGURE S1 | Normalized light intensity autocorrelation functions (a), apparent hydrodynamic radii, R_h, distributions (b), and apparent diffusion coefficients, D_{app}, versus q^2 (c) for sample S1 in a methanol/THF (v:v = 3:1) mixture at a concentration of 0.25 mg/mL.

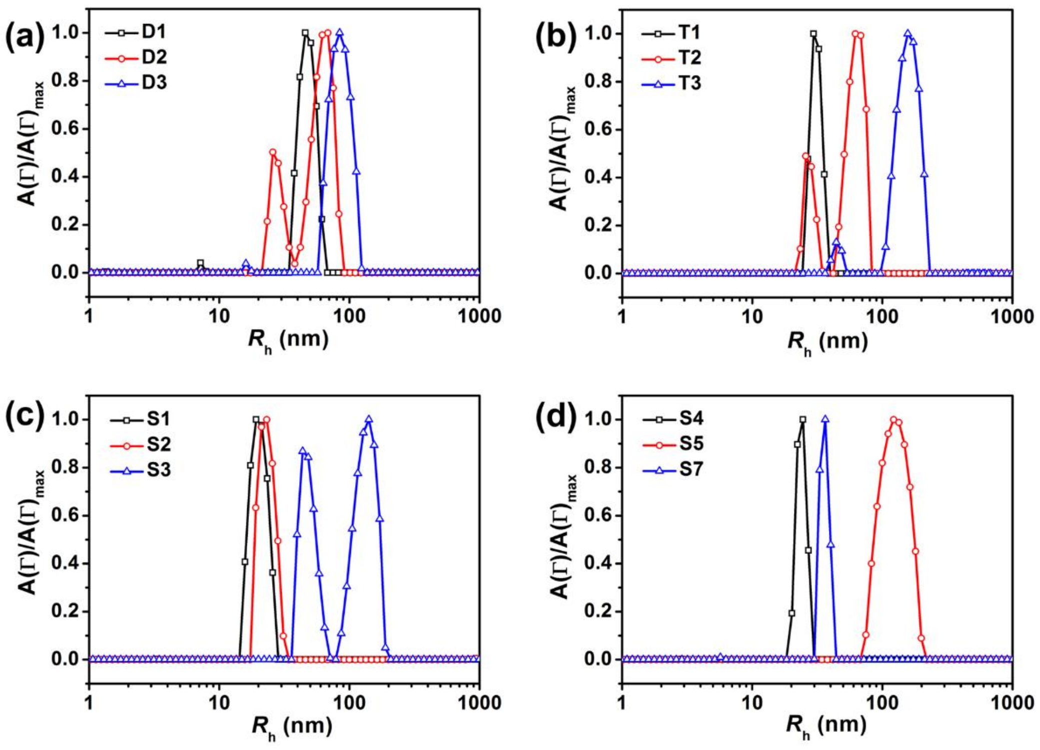

FIGURE S2｜ Representative hydrodynamic radii, R_h, distributions for (a) diblock, (b) triblock, (c) 8-arm star, (d) 26- and 40-arm star copolymers in a methanol/THF (v:v = 3:1) mixture at C = 0.25 mg/mL. The IDs used in the legends of each figure correspond to the sample IDs used in Table 1.

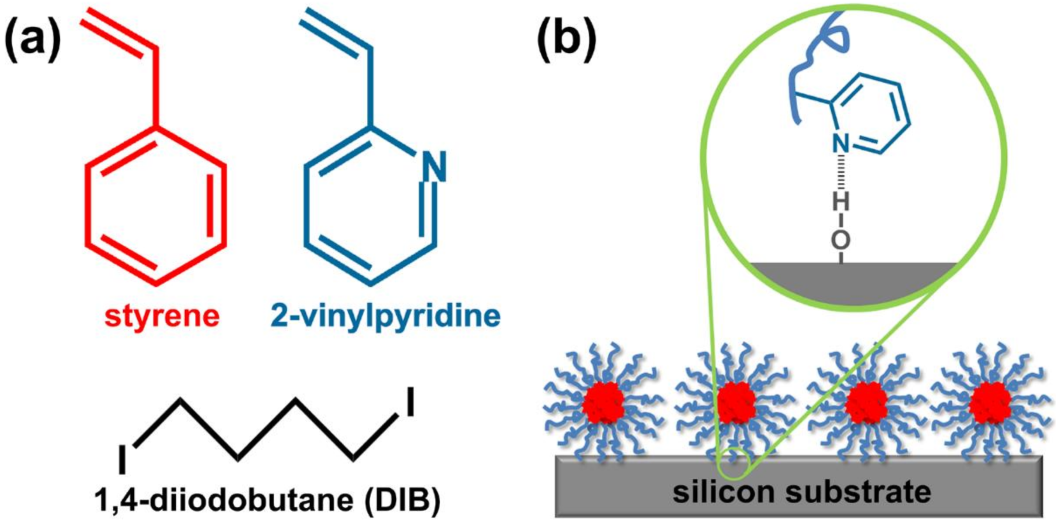

FIGURE S3 | (a) Chemical structures of styrene, 2-vinylpyridine and 1,4-diiodobutane. (b) Schematic illustration of the self-assembly of PS–PVP BCPs on surface, which is promoted by hydrogen bonding interactions between surface silanol groups and the pyridine.

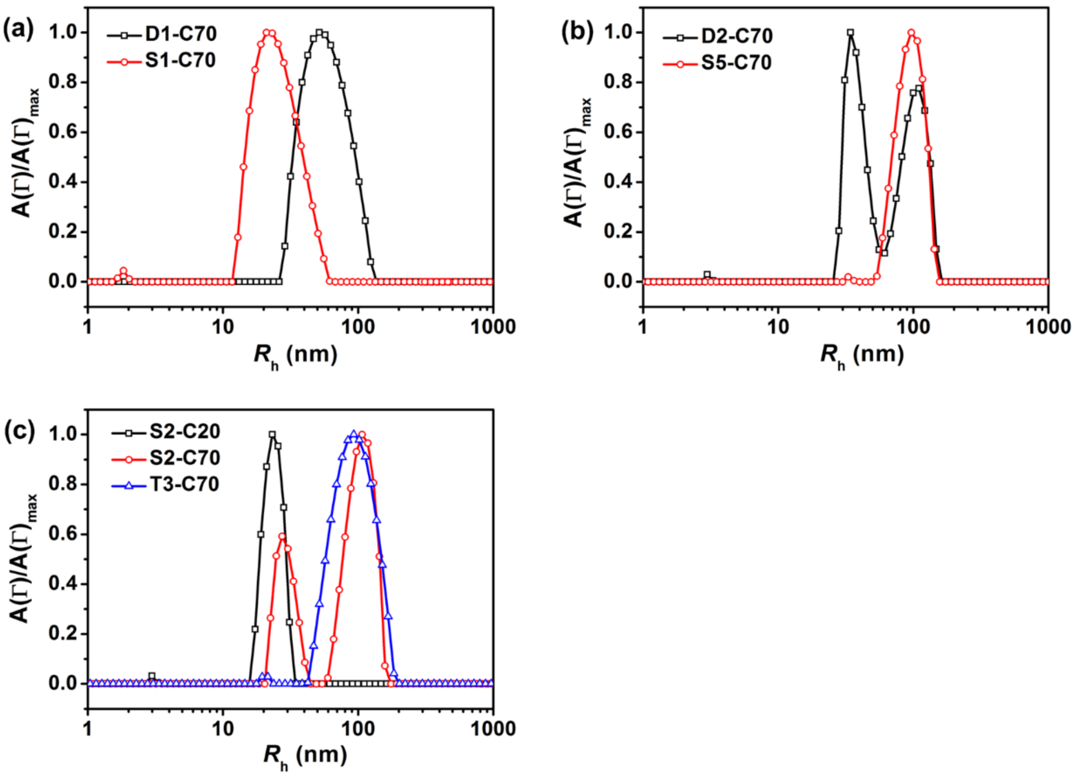

FIGURE S4 | Representative hydrodynamic radii, R_h, distributions for crosslinked BCP NPs in a methanol/THF (v:v = 3:1) mixture at a concentration of 0.25 mg/mL. The IDs used in the legends of each figure correspond to the sample IDs in Table 2.

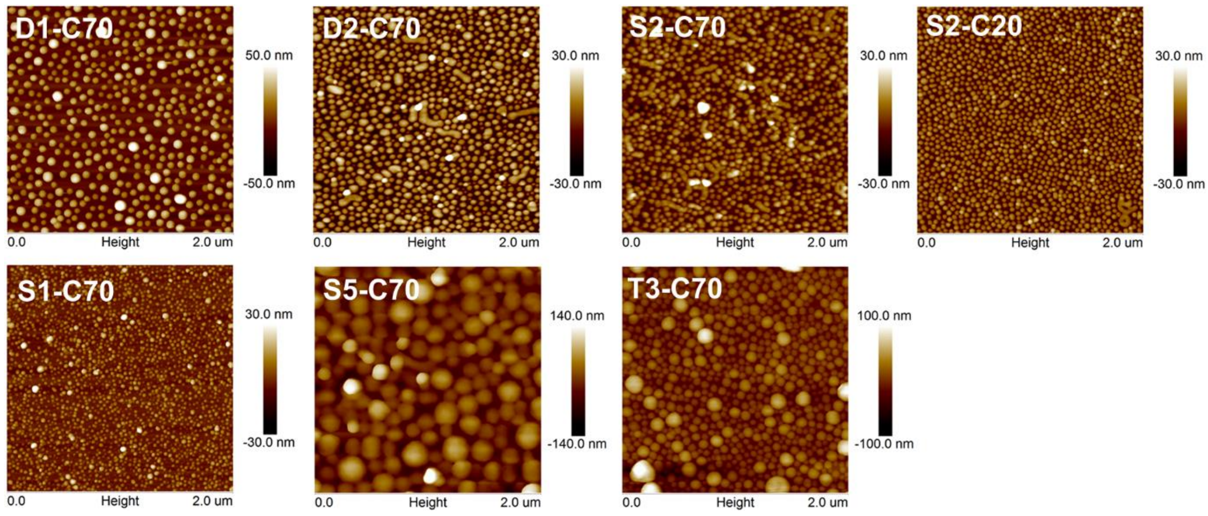

FIGURE S5 | AFM height images (2 µm × 2 µm) of crosslinked BCP NPs. The IDs used in the legends of each figure correspond to the sample IDs given in Table 2.

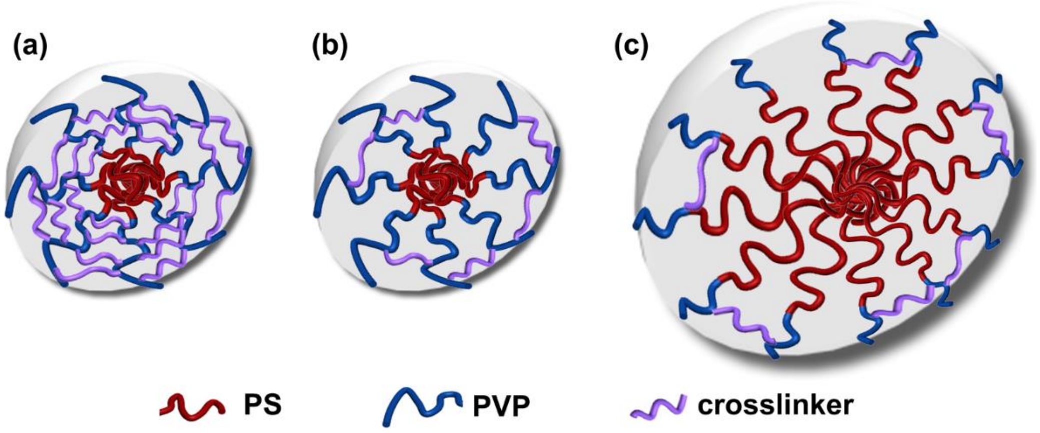

SCHEME S1 | Representations of crosslinked BCP NPs at (a) small S/V ratio and high BCP CLD, (b) small S/V ratio and low BCP CLD, and (c) large S/V ratio and low BCP CLD.
